# Association of player position and functional connectivity alterations in collegiate American football players: an fMRI study

**DOI:** 10.3389/fneur.2024.1511915

**Published:** 2025-01-07

**Authors:** Owen Griffith, Xiaoxiao Bai, Alexa E. Walter, Michael Gay, Jon Kelly, Wayne Sebastianelli, Linda Papa, Semyon Slobounov

**Affiliations:** ^1^Department of Kinesiology, Penn State University, 19 Recreation Building, University Park, PA, United States; ^2^Social, Life, and Engineering Sciences Imaging Center, Social Science Research Institute, Penn State University, 120F Chandlee Laboratory, University Park, University Park, PA, United States; ^3^Department of Neurology, Perelman School of Medicine, University of Pennsylvania, Philadelphia, PA, United States; ^4^Penn State Sports Medicine and Physical Therapy, State College, PA, United States; ^5^Orlando Health, Orlando, FL, United States

**Keywords:** functional connectivity, resting state functional magnetic resonance imaging, American football, default mode network, posterior cingulate cortex, precuneus

## Abstract

**Introduction:**

Resting state-fMRI, provides a sensitive method for detecting changes in brain functional integrity, both with respect to regional oxygenated blood flow and whole network connectivity. The primary goal of this report was to examine alterations in functional connectivity in collegiate American football players after a season of repetitive head impact exposure.

**Methods:**

Collegiate football players completed a rs-fMRI at pre-season and 1 week into post-season. A seed-based functional connectivity method, isolating the posterior cingulate cortex (PCC), was utilized to create individual functional connectivity maps. During group analysis, first, voxel-wise paired sample t-tests identified significant changes in connectivity from pre- to post-season, by player, and previous concussion history. Second, 10 DMN ROIs were constructed by overlaying an anatomical map over regions of positive correlation from one-sample t-tests of pre-season and post-season. These ROIs, plus the LpCun, were included in linear mix-effect modeling, with position or concussion history as covariates.

**Results:**

66 players were included (mean age 20.6 years; 100% male; 34 (51.5%) non-speed position players). The 10 DMN ROIs showed no alterations from pre-season to post-season. By concussion history, the right temporal ROI demonstrated a significant effect on baseline functional connectivity (*p* = 0.03). Speed players, but not non-speed players, demonstrated a significant decrease in functional connectivity in the precuneus from pre- to post-season (*p* < 0.001).

**Discussion:**

There are region-specific differences functional connectivity related to both position and concussion history in American collegiate football players. Player position affected functional connectivity across a season of football. Position-specific differences in head impact exposure rate and magnitude plays a crucial role in functional connectivity alterations.

## 1 Introduction

Repetitive head impact (RHI) exposure in sport is a topic of growing concern in clinical medicine, due to its potentially negative effects on long-term brain health (1–8). Participation in contact sport is a setting in which both RHIs and concussions or mild traumatic brain injuries (mTBI) are common. American Football, in particular, represents the sport with the highest rate of male youth participation in United States and also accounts for the highest rate of reported concussive injuries among U.S. youth sports (9, 10). Historically, RHIs have been defined as subconcussive impacts to the head in which visible signs or symptoms of neurological dysfunction may not develop despite those impacts having the potential for neurological injury (11). However, the long-term negative effects of accumulated RHIs in sport have been previously reported (12) and associations between the accumulation of RHIs over a sport career and downstream neurodegenerative processes have been established (5).

Considering the associations with future neurodegenerative processes, it is critical that tools to measure brain alterations during sport participation are established in clinical practice. Brain alterations associated with functional or behavioral changes in young athletes who are experiencing frequent RHIs can be difficult to measure, especially given these athletes are often young, healthy, active, and a resilient population (13). Brain neuroimaging is a commonly used measure to detect alterations in functional and structural integrity associated with RHIs, despite its current limitations in cost and standardization (14). Functional connectivity between brain regions and networks, or the temporal correlation of regional alterations in the ratio of oxygenated, compared to deoxygenated cerebral blood flow, is measured using functional magnetic resonance imaging (fMRI), and presents a sensitive method for detecting subtle changes in brain health (15). This is due in large part to fMRI’s power to map highly specific changes in regional coordination of neurometabolism in the absence of detectable structural damage to the brain (16).

Functional connections are commonly mapped across functional networks of the brain (17, 18). Naturally, the regions by which these networks are defined, demonstrate highly correlated functional connectivity, with respect to specific and unique brain functions (19). Two well defined networks, which have been studied extensively, using resting state-fMRI (rs-fMRI), for its sensitivity to changes in the brain function after head injuries of varying severity, is the Default Mode Network (DMN) and the Cognitive Control Network (CCN) (20–27) These network have been extensively studied and are sensitive to changes in the brain function after head injuries of varying severity (20–27). The default mode network (DMN) in particular has been extensively examined. Previous studies have demonstrated within-network hyperconnectivity of the DMN after a season of RHI exposure (28–37). However, hypoconnectivity after RHI accumulation has also been demonstrated in athletes (37). There have also been preliminary associations between alterations in within- and between-network connectivity resultant from RHIs and later-in-life neurodegenerative disease (38) following participation in football. However, this link remains largely understudied due to the lack of clarity surrounding network alterations after shorter periods of RHI exposure (i.e., across a single or multiple seasons).

One large confounding factor surrounding lifetime exposure to RHIs is player position on the field. Typically, in American football, players are considered speed (quarterbacks, wide receivers, linebackers, and defensive backs) and non-speed positions (defensive and offensive linemen), based on the role of the player on the field (39, 40). In American football, research using xPatch accelerometers has shown that there are differing rates of RHIs based on player positions (41). Speed positions often require players to engage in aggressive tackles or to be on the receiving end of such tackles, leading to a higher probability of moderate to high velocity head acceleration events (HAE) (42). These players also are often at higher risk for experiencing mTBI (43–45). In contrast, non-speed positions are more consistently involved in head-to-head contact at the line of scrimmage. These positions experience moderate to lower force HAE’s but at a higher frequency (41, 42) and typically have lower rates of mTBI (46). Additionally, work from our group has shown that serum concentrations of blood biomarkers glial fibrillary acidic protein (GFAP) and neurofilament light chain (NFL) increased significantly over the course of a single collegiate football season and that the highest concentrations were in speed positions compared to non-speed positions (47).

Additionally, brain imaging studies have demonstrated football position-specific differences in various MRI metrics following exposure to RHIs. Specifically, defensive backs experienced more concussions and subsequent blood–brain barrier leaking than other positions (48), speed players exhibited a larger number of metabolic changes in dorsolateral prefrontal cortex and M1 metabolites as compared with non-speed players (42), and speed players incurred mainly low cycle fatigue damage profiles of Tau deposition (49). Therefore, player position is an important confounder when analyzing the physiological consequences of exposure to RHIs in currently participating athletes.

Another confounding factor to exposure to RHIs is history of previous concussions (Cx). Cx has been associated with more severe symptom outcomes from subsequent concussions (50–52). Additionally, in regard to MRI work, some studies have found no difference in baseline functional connectivity in individuals with concussion history compared to individual without concussion history (24, 53). However, there is evidence that after a single competitive game involving exposure to RHIs, in this case rugby, those with a history of concussion showed greater alterations in DMN functional connectivity (31).

The overall goal of this study was to explore functional connectivity alterations both within the DMN and between the DMN and other brain regions using rs-fMRI over the course of a single season of collegiate American football. The DMN may show a large-scale, within-network hyperconnectivity response to a season of RHI exposure. However, it is still unclear how within-network or between network connectivity may change after a season of RHIs. Specifically, we aimed to (a) examine alterations in functional connectivity in the DMN from pre- to post-season in a large cohort of American collegiate football players, (b) examine alterations in functional connectivity between the DMN and other brain ROIs from pre- to post-season, and (c) identify if there is an effect of two major covariates, player position and concussion history, on these DMN related measures of functional connectivity.

## 2 Methods

### 2.1 Study design and setting

This prospective observational study was conducted at Penn State University, a public institution of learning and a participating member institution of the Big Ten Conference and NCAA Division I of the National Collegiate Athletic Association (NCAA).

### 2.2 Study population and procedures

Division I NCAA collegiate American football athletes were recruited over 4 years (2015, 2019, 2021, 2022). Inclusion criteria for participation included 18 years or older and participation in intercollegiate athletic contact sport (American football). Exclusion criteria included the existence of a concurrent athletic injury that would preclude them from full participation during the season and contraindications to MRI (such as claustrophobia, metal orthodontic braces, and metal or cochlear implants). This study was approved by the Institutional Review Board of Penn State University and all participants provided written informed consent.

**Figure 1:**
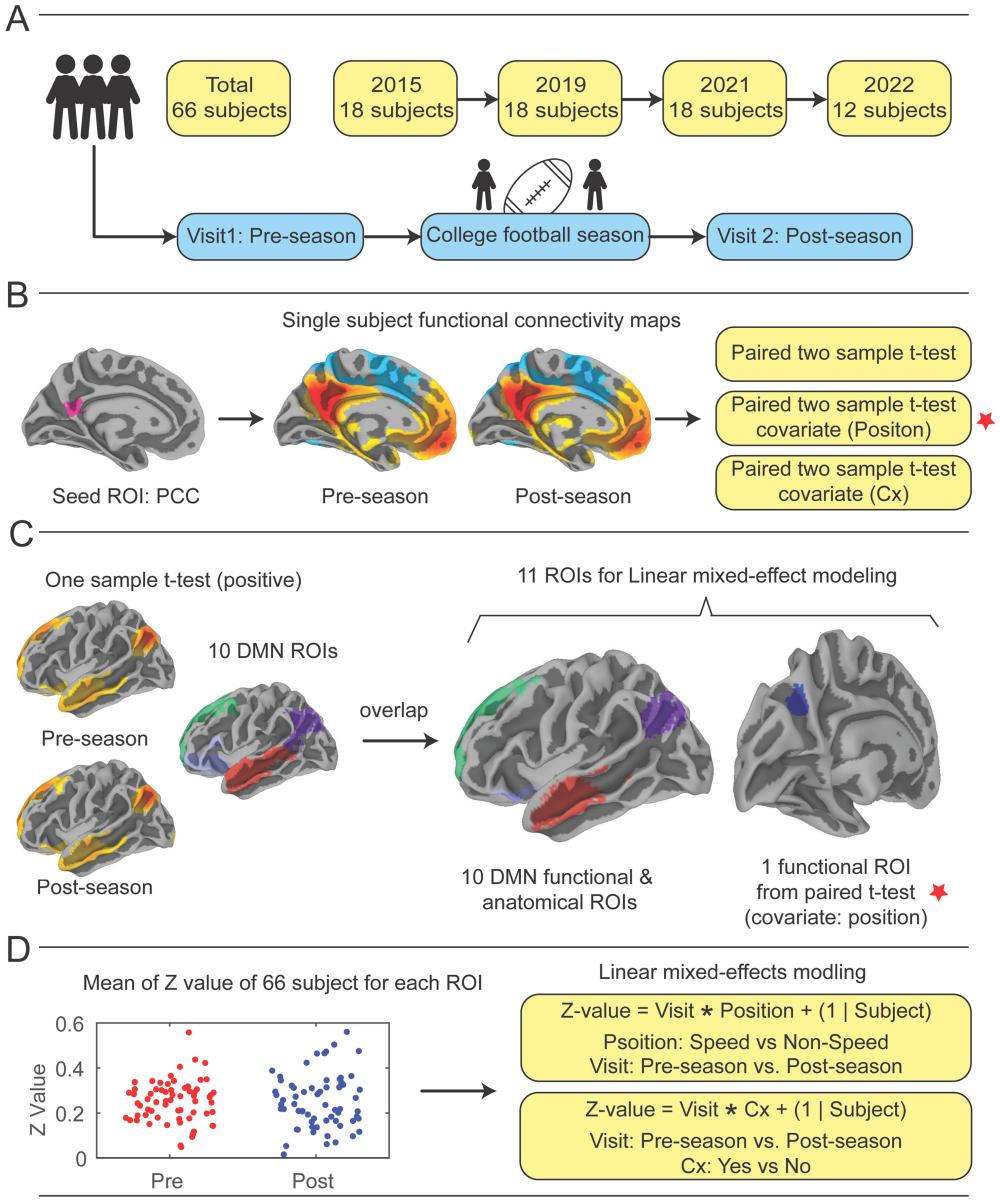
Study design. **(A)** Participants were recruited over the course of four collegiate football seasons. Each year, an identical imaging acquisition protocol was followed, which included a pre-season fMRI scan prior to any hitting practices beginning in the football preseason, the football season of note, and a post-season scan directly after the regular season ended. **(B)** The posterior cingulate cortex (PCC) was pre-selected as a seed region, then correlation coefficients of mean time series of the PCC compared with all other brain voxels were calculated to demonstrate functional connectivity (FC) with the whole brain for pre-season and post-season scans (See Supplementary Figure 2A). Paired-sample t-tests were performed to identify significant differences in functional connectivity from pre- to post-season. Two covariates, player position and previous concussion history (Cx), were included in analysis. **(C)** Ten DMN ROIs were constructed by performing one-sample t-tests on pre- and post-season scans to identify regions that showed positively correlated mean time series with the PCC, then overlaying them upon 10 DMN ROIs from the Schaefer-Yeo atlas (58) (See Supplementary Figure 2B). 10 DMN ROIs and the one significant ROI in the left precuneus (LpCun) were used for linear mixed-effect modeling with covariates. **(D)** Utilizing linear mixed effect modeling with position and concussion history as covariates, the functional connectivity correlation coefficients for pre- and post-season were calculated, then changes from pre- to post-season were calculated by transforming the correlation coefficients to Fisher’s Z-scores to improve normality and subtracting pre-season Z-score from post-season Z-score (See Supplementary Figures 2–6). Cx = concussion.

All participants completed a pre-season interview which included demographic information, and medical and concussion history. Participants were contacted during the summer period of their sport (Figure 1A). At this time, they were taking part in standard non-contact summer strength and conditioning exercises, but not in any type of formal football practice and were not exposed to any head impacts or hitting. Prior to the start of preseason of each respective fall NCAA football season (when hitting practices begin), participants completed one MRI protocol which included a rs-fMRI sequence. During the fall football seasons, all participants included in the study participated in standard team activities, including football practices and games. Participants then completed a post-season MRI scan, which included a rs-fMRI sequence, within 7–14 days immediately following the conclusion of the Fall NCAA regular football season.

### 2.3 MRI acquisition

All imaging data were acquired using a 3 tesla Magnetom Prisma-Fit scanner (Siemens Medical Systems, Erlangen, Germany) with a 20-channel head coil. The imaging protocol included the acquisition of T1-weighted structural images, resting-state blood oxygen level-dependent (BOLD) functional scans (rsfMRI) data, and field maps for distortion correction. Sagittal 3D magnetization-prepared rapid gradient echo (MPRAGE) was acquired using the repetition time (TR) = 1700 ms, echo time (TE) = 1.77 ms, inversion time (TI) = 850 ms, flip angle = 9 degrees, field of view (FOV) = 256 mm x 256 mm, voxel size = 1.0 × 1.0 × 1.0 mm, slices = 178, and scanning time = 3 min 34 s. Functional images were acquired using a 2D multiband echo-planar imaging (EPI) sequences (Release R016a, from the Center for Magnetic Resonance Research (CMRR), University of Minnesota), with a TR = 2000 ms, TE = 35.8 ms, flip angle = 90 degrees, FOV = 192 mm x 192 mm, voxel size = 2.0 × 2.0 × 2.0 mm, slices = 72, multiband celebration factor = 3, and 300 repetition times (scanning duration of 10 min 10 s). To correct for susceptibility-induced distortions in the EPI images, field maps were acquired using a dual-echo gradient echo sequence (TEs = 4.92/7.38 ms). The individuals were instructed to lie awake with their eyes open. Of the original 117 participants recruited and consented at preseason, a total of 51 were removed prior to analysis as a result of increased body mass over the season. Twenty-eight of these removed were unable to complete their post-season scan due to increased body mass since preseason, that was incompatible with physically entering the scanner. An additional 23 participants’ data were removed due to motion artifact on their postseason scan, which was related to inability to comfortably fit within the standard 60 cm bore and 64-channel head coil used in this study.

### 2.4 MRI preprocessing

The preprocessing of the resting-state fMRI data was performed using fMRIPrep (version 23.1.0) (54), a standardized preprocessing pipeline for fMRI data. The preprocessing steps included motion estimation, slice timing correction, susceptibility distortion correction, co-registration of functional to anatomical images, and spatial normalization to the MNI152NLin2009cAsym standard space.

The smoothness and denoising procedure were performed by using AFNI (version 21.3.04). Spatial smoothing using gaussian kernel of 6 mm full-width half-maximum was implemented. To further reduce noise related to motion and physiological fluctuations, a general linear model (GLM) was used to perform additional denoising steps. Motion and physiological noise were addressed through nuisance regression calculated by fMRIPrep, which included six motion parameters (three translations and three rotations), their first derivatives, global signal regressor, and seven anatomical CompCor regressors (55, 56). A total of 20 regressors were included in the general linear model (GLM) to account for motion and physiological noise, ensuring that only the neural signals of interest remain. Frames that exceeded a threshold of 0.6 mm framewise displacement were annotated as motion outliers (57). The functional image data was bandpass filtered between 0.01 and 0.1 Hz.

### 2.5 Functional connectivity analysis

Functional connectivity analysis was performed by using AFNI (version 21.3.04). A seed region in the posterior cingulate cortex (PCC), (right and left pCunPCC_1, from the 400 parcels of the 7 networks defined by the Schaefer-Yeo atlas) (58) was used to create individual functional connectivity maps. The PCC was chosen due to its critical role in the default mode network (DMN), serving as a central hub of the DMN (59–61), and its previous applications in concussion studies (30, 62, 63). The mean time series from the PCC was extracted and correlated with the time series of all other voxels in the brain. Pearson correlation coefficients were calculated and transformed to Fisher’s Z-scores to improve normality.

Group-level statistical analysis was conducted using two separate approaches. In the first analysis, voxel-wise paired t-test (Figure 1B) was performed to assess differences in connectivity patterns between pre-season and post-season by using AFNI’s 3dttest++. Player position and Cx were added as covariates for the pre-season and post-season test. The results of the paired t-tests were corrected for multiple comparisons using a cluster-based thresholding approach (AFNI’s Clustsim) with a significance level of *p* < 0.05 (uncorrected *p* < 0.001, cluster size = 134 voxels, voxel size = 2.00×2.00×2.00 mm^3) (Figure 1B).

In addition to the whole-brain seed-to-voxel analysis, a second ROI-based analysis was conducted to examine group differences in mean Z-values within 10 DMN ROIs and one ROI identified as significant from the paired t-tests (Figure 1B). In the analysis of 11 ROIs, including one ROI identified from the voxel-wise paired t-test and 10 atlas-based DMN ROIs, no correction for multiple comparisons was applied. This analysis was exploratory in nature, and results should be interpreted with caution. Future studies may benefit from applying correction methods, such as FDR or FWE, to address multiple testing. The 10 DMN ROIs were constructed by combining the 24 DMN ROIs from the Schaefer-Yeo atlas with group-clustered results derived from one-sample analyses.

First, 10 atlas-based DMN ROIs were derived from the 24 DMN ROIs in the Schaefer-Yeo atlas (spanning 7 networks and 100 regions) (58). These ROIs were constructed as follows (Supplementary Figure 1): (1) left temporal (Default_Temp_1 + Default_Temp_2); (2) left parietal (Default_Par_1 + Default_Par_2); (3) left prefrontal cortex PFCdPFCm (Default_PFC_1 + Default_PFC_2); (4) left PFCv (Default_PFC_3 + Default_PFC_4 + Default_PFC_5 + Default_PFC_6 + Default_PFC_7); (5) left pCuPCC (Default_pCunPCC_1 + Default_pCunPCC_2); (6) right parietal PAR (Default_Par_1); (7) right temporal (Default_Temp_1 + Default_Temp_2 + Default_Temp_3); (8) right PFCv (Default_PFCv_1 + Default_PFCv_2); (9) right PFCdPFCm (Default_ PFCdPFCm _1 + Default_ PFCdPFCm _2 + Default_ PFCdPFCm _3); (10) right pCuPCC (Default_pCunPCC_1 + Default_pCunPCC_2). Second, separate one-sample t-tests were performed on pre-season and post-season scans to identify regions positively correlated with the PCC, using a threshold of corrected *p* < 0.05 (based on uncorrected *p* < 0.001 with a cluster size of 134 voxels). These analyses highlighted well-established DMN areas showing positive correlations with the PCC during task-negative states. Third, the positive regions from the one-sample t-tests for pre-season and post-season scans were overlapped, ensuring that the resultant mask regions were significantly positively correlated with the PCC during both visits. Finally, the 10 atlas DMN ROIs were overlaid with the positive clustered regions to produce the final 10 DMN ROIs, as illustrated in Supplementary Figure 1. Additionally, an extra ROI in the left precuneus (LpCun) was created based on a significant region identified in the paired t-test (voxel count: 202; volume: 1616 mm^3^; MNI peak coordinates: −12.5, −70.5, 35.5) (Figure 1C, marked by red stars).

Group differences in functional connectivity within these ROIs were assessed using an uncorrected linear mixed-effects models implemented in MATLAB (R2022b) The linear mixed effect models were not corrected using not corrected using FDR or FWE methods commonly applied in ROI-based studies, since they acted as a second measure of accuracy to confirm the seed-to-whole-brain voxel analysis, which had already been corrected using the ClustSim method. Two models were used to test group differences (Figure 1D). The first model was defined as:


$$Z-\mathrm{value}=\mathrm{Visit}*\mathrm{Position}+\left(1|\mathrm{Subject}\right)$$

Where Position indicates Speed vs. Non-Speed and Visit indicates Pre-season vs. Post-season.

The second model was defined as:


$$Z-\mathrm{value}=\mathrm{Visit}*Cx-\mathrm{History}+\left(1|\mathrm{Subject}\right)$$

Where Cx indicates History of Yes vs. History of No and Visit indicates Pre-season vs. Post-season (Figure 1D).

## 3 Results

### 3.1 Demographic and outcome characteristics

117 players consented into the study over the four seasons. After removing participants with missing data (*n* = 28) or individuals with non-useable fMRI sequences (*n* = 23), 66 participants were included in analyses. These individuals were a mean age of 20.55 ± 1.52 years, 100% male, and had played football for a mean of 10.84 ± 4.85 years (Table 1). 33% reported at least one previously diagnosed concussion and 48% were considered speed position players. By season, participants did not differ significantly in any demographic factor.

**Table 1:** Participant demographics.

Descriptive variable	All years		2015		2019		2021		2022	
Sample size, *N*	66		18		18		18		12	
Age (yrs), *mean sd*	20.55	1.52	20.94	1.66	20.94	1.55	20.28	1.27	19.75	1.36
Duration played sport (yrs), *mean sd*	10.84	4.85	11.06	3.68	11.22	3.92	10.56	3.65	10.42	4.62
Body type, *mean sd*										
Height (in)	74.35	2.36	75.06	2.10	74.28	2.67	73.94	2.24	74.34	2.45
Weight (lbs)	252.18	47.56	271.83	40.33	247.72	45.55	247.44	55.36	236.5	44.04
Sex, *n* (%)										
Male	66	100.00	18	100.00	18	100.00	18	100.00	12	100.00
Concussion History, *n* (%)										
Previous concussion(s)	22	33.33	8	44.44	7	38.89	4	22.22	3	25.00
No Previous concussion(s)	44	66.67	10	55.56	11	61.11	14	77.78	9	75.00
Position, *n* (%)										
Speed	32	48.48	7	38.89	9	50.00	7	38.89	9	75.00
Non-Speed	34	51.52	11	61.11	9	50.00	11	61.11	3	25.00

### 3.2 Whole-brain, voxel-wise analysis

#### 3.2.1 Seed-based mapping

The seed region PCC demonstrated significant effect of player position in the whole brain voxel-wise paired t-tests. Threshold of corrected *p* < 0.05 (based on uncorrected *p* < 0.001 with a cluster size of 134 voxels) (Supplementary Figure 1). One additional voxel cluster, the LpCun demonstrated significant connectivity with the PCC from the paired t-tests (*p* < 0.01). The results of the ROI analysis are presented as exploratory findings and have not been corrected for multiple comparisons. While this approach provides additional insights, it is important to note that these findings should be validated in future studies using stricter correction methods.

#### 3.2.2 Linear mixed effects model

**Figure 2:**
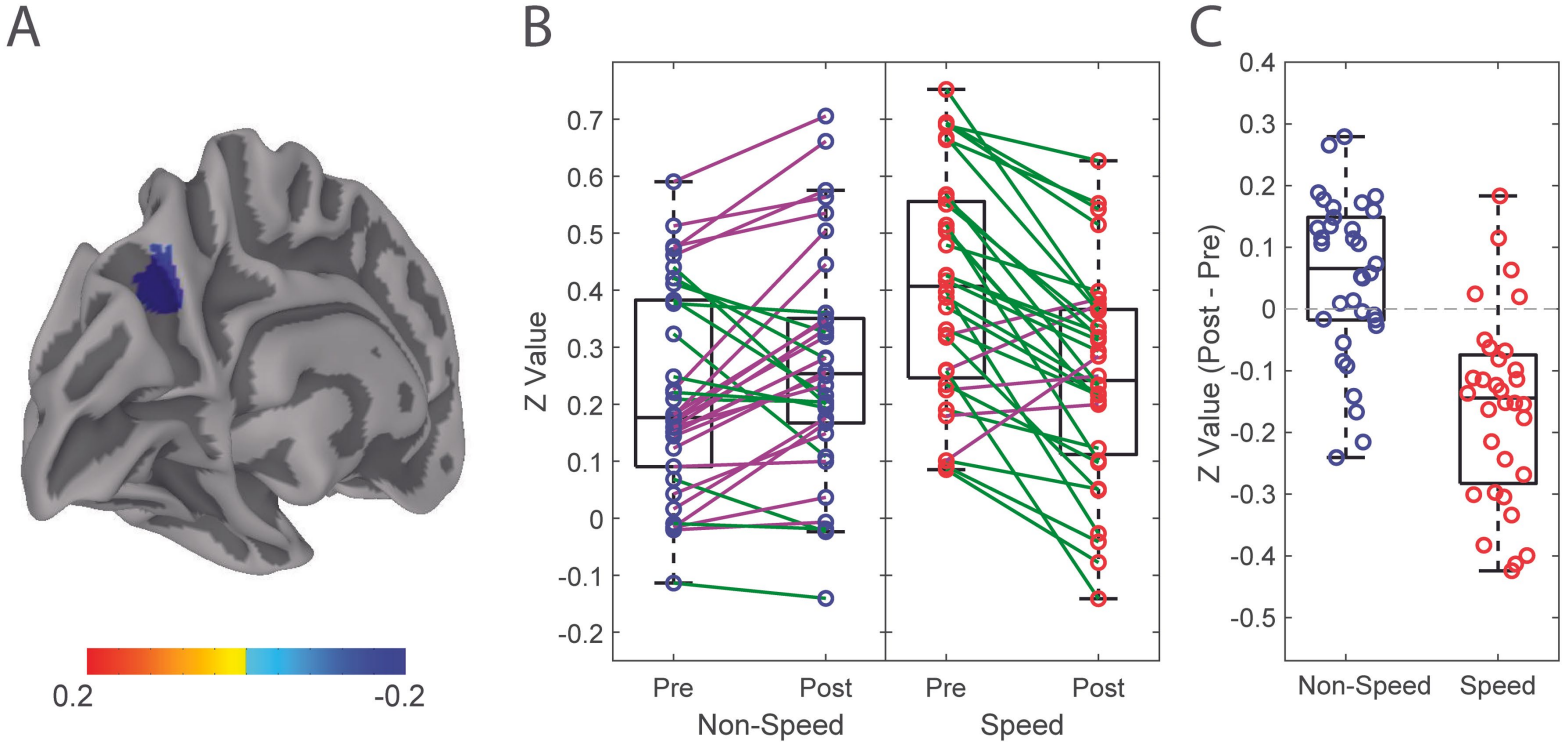
Alteration in functional connectivity from pre-season to post-season of the Left Precuneus in Speed and Non-Speed players. **(A)** Linear mixed effect modeling was used to determine significant differences in functional connectivity correlation of the LpCun from pre-season to post-season, including position type and concussion history (Cx) as a covariate. **(B)** Directionality of alterations in functional connectivity from pre-season to post-season are illustrated in Speed (red) and Non-Speed (blue) players. Positive trend lines indicate increased coherence from pre-season to post-season, while negative trend lines indicated a decrease. **(C)** Difference in functional connectivity between Speed and Non-speed players from pre-season to post-season are further demonstrated using box-and-whisker plots. Negative values indicate decreases in functional connectivity from pre-season to post-season, while positive values indicate increases.

By player position, there was a significant effect of time (*F* (1, 132) = 4.63, *p* = 0.03) and time by position interaction (*F* (1, 132) = 36.95, *p* < 0.001) where speed players had decreased functional connectivity of the LpCun from pre-season to post-season compared to non-speed players (Figure 2). When examining alterations from pre- to post-season in functional connectivity of the LpCun by Cx, there were no significant findings (Table 2).

**Table 2:** Linear mixed-effect model of DMN ROIs: Timepoint x Concussion History.

	Terms					
Brain ROI, *F-*stat *p-*value	Timepoint		Concussion history		Timepoint x Concussion history	
Significant ROI from seed-based analysis						
Left precuneus	6.02	0.02	0.09	0.77	0.84	0.36
Left hemisphere						
Temporal	0.07	0.79	3.00	0.09	0.00	0.95
Parietal	0.03	0.85	0.00	0.97	2.54	0.11
Ventral prefrontal cortex	0.34	0.56	0.02	0.90	1.13	0.29
Precuneus posterior cingulate cortex	0.85	0.36	0.79	0.38	0.95	0.33
Dorsolateral and dorsomedial prefrontal cortex	0.19	0.67	1.01	0.32	0.00	0.95
Right hemisphere						
Temporal	0.30	0.58	**5.04**	**0.03**	0.17	0.68
Parietal	0.41	0.52	0.78	0.38	0.17	0.68
Ventral prefrontal cortex	0.15	0.70	1.95	0.17	0.28	0.60
Precuneus posterior cingulate cortex	0.00	1.00	0.73	0.39	0.99	0.32
Dorsolateral and dorsomedial prefrontal cortex	0.22	0.64	0.57	0.45	0.28	0.60

Significant items are bolded.

### 3.3 Ten default mode network regions of interest

**Figure 3:**
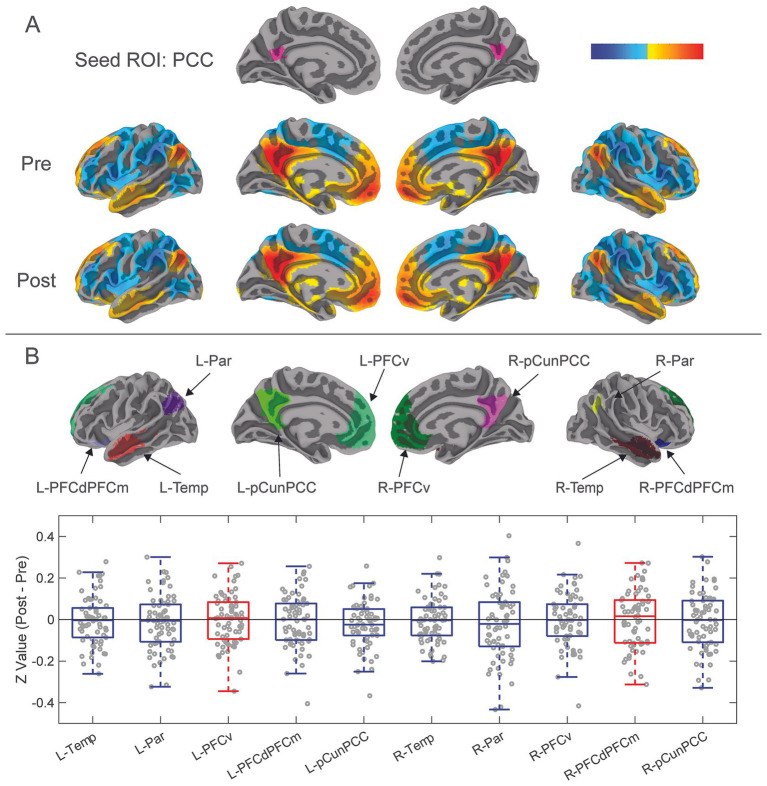
Results from functional connectivity analysis 10 DMN ROIs. **(A)** The PCC was previously isolated as a seed ROI for functional connectivity analysis with the whole brain. Correlation coefficients of mean time series of the PCC compared with all other brain voxels were calculated to demonstrate functional connectivity between the PCC and whole brain for pre-season and post-season scans. Red-yellow areas represent voxels that demonstrated positive correlation coefficients, while blue areas represent negative correlation coefficients. **(B)** Ten additional DMN ROIs were constructed from the combination of the DMN ROIs of the Schaefer-Yeo atlas and group clustered results from one sample t-tests (See Figure 1C; Supplementary Figure 1). DMN ROIs included left and right: temporal, parietal, ventral prefrontal cortex, dorsolateral and dorsomedial prefrontal cortices, and precuneus-posterior cingulate cortex. Difference in pre-season to post-season functional connectivity of the ten DMN ROIs was calculated by transforming their mean time series correlation coefficients to Fisher’s Z-values, then subtracted pre-season from post-season. These pre- to post-season changes are represented with the box-and-whisker plot showing Z-values of paired sample t-tests of post-season minus pre-season functional connectivity of the 10 DMN ROIs. Blue boxes show decreases in hypoconnectivity (decreased functional connectivity) from pre-season to post-season, while Red boxes show hyperconnectivity (increased functional connectivity) from pre-season to post-season.

Functional connectivity changes from pre-season to post-season in ten DMN ROIs were calculated (Figure 3) (Supplementary Figure 2). There were no significant differences in functional connectivity from pre- to post-season within any of these ROIs (Figure 3B). Visually, changes in functional connectivity across the season (post-season minus pre-season) between the PCC and the 10 DMN ROIs demonstrated both hypoconnectivity (8 of the ROIs) and hyperconnectivity (2 of the ROIs).

When examining the influence of player position and Cx on changes in functional connectivity in DMH ROIs, linear mixed models were used to examine functional connectivity alterations from pre- to post-season. There were no significant differences in functional connectivity from pre- to post-season in any of the DMN ROIs by player position (Table 3; Supplementary Figures 3, 4). Concussion history did have a signification effect on functional connectivity, specifically in the right temporal ROI of the DMN (*F* (1,132) = 5.04, *p* = 0.03; Table 2) However, there was no significant effect of timepoint.

**Table 3:** Linear mixed-effect model of DMN ROIs: Timepoint x Position.

	Terms					
Brain ROI, *F, p-*value	Timepoint		Position		Timepoint x Position	
Significant ROI from seed-based analysis						
Left precuneus	**4.63**	**0.03**	0.24	0.63	**36.95**	**0.00**
Left hemisphere DMN ROIs						
Temporal	0.52	0.47	0.13	0.72	0.44	0.51
Parietal	0.54	0.46	0.70	0.40	0.07	0.80
Ventral prefrontal cortex	0.02	0.90	0.05	0.83	0.03	0.86
Precuneus posterior cingulate cortex	0.66	0.42	0.03	0.87	0.35	0.56
Dorsolateral and dorsomedial prefrontal cortex	0.00	0.96	0.03	0.86	0.24	0.62
Right hemisphere						
Temporal	0.78	0.38	0.20	0.66	0.75	0.39
Parietal	2.09	0.15	1.65	0.20	1.03	0.31
Ventral prefrontal cortex	0.23	0.63	0.06	0.80	0.67	0.42
Precuneus posterior cingulate cortex	0.37	0.54	0.67	0.42	0.05	0.82
Dorsolateral and dorsomedial prefrontal cortex	0.06	0.81	0.04	0.84	0.01	0.91

Significant items are bolded.

## 4 Discussion

In this observational study, we examined changes of functional connectivity in a relevant and well-documented neural network, the Default Mode Network. Several key findings were highlighted in this study. First, functional connectivity within the functional ROIs of the DMN, remained largely unchanged from pre-season to post-season, a period during which participants were exposed to high accumulation of RHIs. Second, the inclusion of player position and concussion history as covariates had varying effects on functional connectivity changes from pre- to post-season, with the most tangible finding being the significant decrease in functional connectivity of the LpCun in Speed, but not Non-Speed players.

### 4.1 Ten default mode network regions of interest

There were no significant alterations in functional connectivity observed from pre- to post-season within the 10 DMN ROIs. This finding was contradictory to our initial hypothesis, as much of the literature has identified the DMN as a network that is susceptible to alterations in functional connectivity after RHI (21, 28, 30, 35). The findings here showed both non-significant hyper- and hypo-connectivity across the season in different ROIs. This finding, paired with inconsistencies in the directionality of DMN functional connectivity changes in studies that did show alterations, may suggest that the DMN is less susceptible to consistent specific alterations after RHI than it is after reported mTBI. Additionally, other networks may be more reliable targets for acquiring consistent results with respect to alterations after RHI.

With respect to the finding of significant group difference in functional connectivity of the right temporal ROI of the DMN, there was a significant effect of concussion history, but no significant effect of timepoint. There are mixed findings in the literature surrounding alterations in functional connectivity as a result of concussion history, with some studies reporting no differences (24, 64) and others reporting altered baseline functional connectivity (65, 66) in those with a history of concussion. However, the mechanism through which functional connectivity alterations occurred in the unilateral temporal region specifically is unknown and may be explored further in future research.

### 4.2 Single season functional connectivity changes of the LpCun are dependent on player position

Player position was associated with alterations in functional connectivity from pre-season to post-season. Speed players demonstrated a decrease in functional connectivity from pre-season to post-season, while non-speed players showed no differences. There are biomechanical differences in the magnitude, location, and frequency of RHIs between the two position groups. Speed players receive less RHIs, with greater average force per RHI, and at varying locations across the helmet, however Non-speed players tend to receive more RHIs with less average force per RHI, and localized RHIs to the crown and forehead, a location associated with lower neural tissue strain (40, 47, 67). This finding may suggest that, as a result of the different biomechanical signatures of the RHIs, speed players may be at greater risk for alterations in brain health after a single season, compared with non-speed players. This imaging finding, although mechanistically unique, is complimentary to previous work from our group that showed significantly higher serum concentrations of TBI biomarkers in Speed, compared to Non-speed players after a single collegiate football season, regardless of whether a concussion was incurred during a season (47).

The precuneus is a region integrated with both the DMN (68, 69) and CCN (69–71), and because the precuneus has been shown to play a major role in integrating high level brain functions such as attention (72, 73) and visual and auditory memory (74), this finding may suggest a greater risk of decreased capacity of these functions. However, this finding should be carefully considered in context as other research shows that non-speed players are at greatest risk of early neurodegenerative processes (75). It may be that the timeframe being considered is critical when examining player position. The overall accumulation of RHIs over a lifetime is a known risk for neurodegenerative disease (5). However, alterations in short-term (i.e., over a single season) outcomes may be more affected by higher impact RHIs, like those received in speed positions. The specific connection between the LpCun and PCC should be of particular note for future examination of functional connectivity alterations after RHIs.

Cx had no relationship with alterations in functional connectivity of the LpCun from pre-season to post-season. This finding is consistent with our previous TBI biomarker work in the same population (76). Of note, however, Cx can influence symptom outcomes. The number of previous concussions and the time since last concussion can affect severity and symptom profile of future concussions (50–52). Additionally, concussion history has long been implicated as a risk factor for early onset of neurodegenerative processes (77). However, there are conflicting results about the effect of previous concussion on outcomes of RHI accumulation. Several studies have shown no neural signatures of alterations in functional connectivity related to concussion history in young or middle-aged individuals (24, 53), while our group has previously demonstrated clear concussion history-dependent alterations in connectivity after a single game of RHI exposure in rugby, in the DMN (31). Guidelines developed at the first Safety in College Football Summit in 2014, which enforced a decrease in opportunities for RHI accumulation over the calendar year may have played a role in the lack of functional connectivity alterations related to concussion history (78). Perhaps the lack of effect of concussion history in this study is due to the relative good health and fitness of our younger population (13). It appears that concussion history may act as a risk factor for alterations in brain health later in life but may not be a fully conclusive marker of subtle early life deficits, particularly in otherwise healthy populations.

### 4.3 Limitations

This study was subject to several inherent limitations, including those related to sample characteristics, imaging acquisition, brain parcellation, and quantification of covariates. Our sample consisted exclusively of male participants as a function of the sport of interest (American football). However, the omission of female participants does detract from the generalizability of our findings as we cannot include sex as a biological covariate. Additionally, due to the altered body composition (increased mass) of our sample population, neuroimaging acquisition presented an administrative challenge to fit particular individuals comfortably in the 3 T Tesla scanner at postseason. Thus, we were forced to remove participants as a result of incomplete or unsuccessful postseason scans, related to inability to fit in the scanner and movement artifact, respectively. With respect to brain map parcellation, the use of the Schaefer 400 atlas comes with inherent pitfalls due to its universality. However, this blanket application of a single atlas was accounted for by overlaying individual functional connectivity maps from the seed-based method. Given the focus on cortico-cortical connectivity, we also recognize that our volume-based approach offers imperfect localization of function to precise cortical anatomy. Furthermore, our two covariates of interest, position and Cx, were imperfect for different reasons. Position, while a validated determinant of biomechanical characteristics of RHI exposure type, does not provide as comprehensive a measure of RHI accumulation as would helmet accelerometer data. Cx was collected using a self-report questionnaire, thus may not have been a completely valid record of medically diagnosed concussions for all participants. It is important to note that none of the participants included in this study were diagnosed with a concussion by medical personnel during the course of the season. Additionally in contact sports, concussion often goes undiagnosed, thus this measure of Cx may inherently lack some degree of accuracy.

## 5 Conclusion

In a 4-season cohort of collegiate American football players, functional connectivity of specific regions and networks are affected differently after a single season of exposure to RHIs. Player position is an important covariate to consider when assessing functional connectivity alterations, especially in the LpCun. There were no significant alterations in the 10 DMN ROIs across the season, despite ROIs showing either hyper-connectivity or hypo-connectivity from pre- to post-season. These findings suggest that brain alterations, as assessed by fMRI, after a single season of RHIs are possible and certain players may be more susceptible to these changes.

### 5.1 Transparency, rigor and reproducibility summary

A minimum sample size of 32 participants, with a maximum sample of size of 120, was planned based on availability of participants from the sample population, a highly specific player position types from a Division-1 American football team, during a specific annual time point. A maximum sample size of 120 participants was planned based upon funding capabilities. A sample size of 117 participants were initially screened, and useable imaging data were obtained and analyzed from 66. Imaging data were obtained between August 2015 and December 2022. Imaging acquisition and analyses were performed by team members blinded to relevant characteristics of the participants, and clinical outcomes were assessed by team members blinded to imaging results. All equipment and software used to perform imaging and preprocessing are widely available from commercial sources. Statistical analysis and/or review was performed by Bai, X., who holds the title of Technical Co-Director of the Penn State University Social, Life, and Engineering Sciences Imaging Center. All equipment and software used to perform imaging and preprocessing are widely available from commercial sources. No replication or external validation studies have been performed or are planned/ongoing at this time to our knowledge.

## Data Availability

The raw data supporting the conclusions of this article will be made available by the authors, without undue reservation.

## References

[1] Bazarian JJ Zhu T Zhong J Janigro D Rozen E Roberts A. Persistent, long-term cerebral white matter changes after sports-related repetitive head impacts. PLoS One. (2014) 9:e94734. doi: 10.1371/journal.pone.0094734, PMID: 24740265 PMC3989251

[2] Di Battista AP Rhind SG Richards D Churchill N Baker AJ Hutchison MG. Altered blood biomarker profiles in athletes with a history of repetitive head impacts. PLoS One. (2016) 11:e0159929. doi: 10.1371/journal.pone.0159929, PMID: 27458972 PMC4961456

[3] Lyon L. High impact research: investigating the effects of repetitive head injury. Brain. (2017) 140:e6. doi: 10.1093/brain/aww294, PMID: 28031226

[4] McAlister KL Mack WJ Bir C Baron DA Som C Li K. Longitudinal, prospective study of head impacts in male high school football players. PLoS One. (2023) 18:e0291374. doi: 10.1371/journal.pone.0291374, PMID: 37682984 PMC10490840

[5] McKee AC Cantu RC Nowinski CJ Hedley-Whyte ET Gavett BE Budson AE. Chronic traumatic encephalopathy in athletes: progressive tauopathy after repetitive head injury. J Neuropathol Exp Neurol. (2009) 68:709–35. doi: 10.1097/NEN.0b013e3181a9d503, PMID: 19535999 PMC2945234

[6] McKee AC Stein TD Nowinski CJ Stern RA Daneshvar DH Alvarez VE. The spectrum of disease in chronic traumatic encephalopathy. Brain. (2013) 136:43–64. doi: 10.1093/brain/aws307, PMID: 23208308 PMC3624697

[7] McKee AC Alosco M Huber BR. Repetitive head impacts and chronic traumatic encephalopathy. Neurosurg Clin N Am. (2016) 27:529–35. doi: 10.1016/j.nec.2016.05.009, PMID: 27637402 PMC5028120

[8] Strauss SB Fleysher R Ifrah C Hunter LE Ye K Lipton RB. Framing potential for adverse effects of repetitive subconcussive impacts in soccer in the context of athlete and non-athlete controls. Brain Imaging Behav. (2021) 15:882–95. doi: 10.1007/s11682-020-00297-4, PMID: 32712797 PMC7861653

[9] Mueller FO Colgate B. Annual survey of football injury research 1931–2010. The National Center for Catastrophic Sport Injury Research (2021)

[10] O’Connor KL Baker MM Dalton SL Dompier TP Broglio SP Kerr ZY. Epidemiology of sport-related concussions in high school athletes: National Athletic Treatment, injury and outcomes network (NATION), 2011-2012 through 2013-2014. J Athl Train. (2017) 52:175–85. doi: 10.4085/1062-6050-52.1.15, PMID: 28387555 PMC5384816

[11] Bailes JE Petraglia AL Omalu BI Nauman E Talavage T. Role of subconcussion in repetitive mild traumatic brain injury. (2013); Available at:https://thejns.org/view/journals/j-neurosurg/119/5/article-p1235.xml 10.3171/2013.7.JNS121822 23971952

[12] Casper ST. Concussion: a history of science and medicine, 1870-2005. Headache J Head Face Pain. (2018) 58:795–810. doi: 10.1111/head.13288, PMID: 29536502

[13] Walter A Herrold AA Gallagher VT Lee R Scaramuzzo M Bream T. KIAA0319 genotype predicts the number of past concussions in a division I football team: a pilot study. J Neurotrauma. (2019) 36:1115–24. doi: 10.1089/neu.2017.5622, PMID: 30351182

[14] Marek S Tervo-Clemmens B Calabro FJ Montez DF Kay BP Hatoum AS. Reproducible brain-wide association studies require thousands of individuals. Nature. (2022) 603:654–60. doi: 10.1038/s41586-022-04492-9, PMID: 35296861 PMC8991999

[15] Mier W Mier D. Advantages in functional imaging of the brain. Front Hum Neurosci. (2015) 9:249. doi: 10.3389/fnhum.2015.00249, PMID: 26042013 PMC4436574

[16] Rogers BP Morgan VL Newton AT Gore JC. Assessing functional connectivity in the human brain by FMRI. Magn Reson Imaging. (2007) 25:1347–57. doi: 10.1016/j.mri.2007.03.007, PMID: 17499467 PMC2169499

[17] Friston KJ. Functional and effective connectivity in neuroimaging: a synthesis. Hum Brain Mapp. (1994) 2:56–78. doi: 10.1002/hbm.460020107, PMID: 39699678

[18] van den Heuvel MP Hulshoff Pol HE. Exploring the brain network: a review on resting-state fMRI functional connectivity. Eur Neuropsychopharmacol. (2010) 20:519–34. doi: 10.1016/j.euroneuro.2010.03.008, PMID: 20471808

[19] Park HJ Friston K. Structural and functional brain networks: from connections to cognition. Science. (2013) 342:1238411. doi: 10.1126/science.1238411, PMID: 24179229

[20] Abbas K Shenk TE Poole VN Breedlove EL Leverenz LJ Nauman EA. Alteration of default mode network in high school football athletes due to repetitive subconcussive mild traumatic brain injury: a resting-state functional magnetic resonance imaging study. Brain Connect. (2015) 5:91–101. doi: 10.1089/brain.2014.0279, PMID: 25242171

[21] Abbas K. Effects of concussive and repetitive subconcussive injury in high school football athletes using resting state FMRI [internet] [Ph.D.]. Dissertation abstracts international: Section B: The sciences and engineering. Purdue University; (2016). Available at:https://ezaccess.libraries.psu.edu/login?url=https://www.proquest.com/dissertations-theses/effects-concussive-repetitive-subconcussive/docview/1930470701/se-2?accountid=13158

[22] Abdul Rahman MR Abd Hamid AI Noh NA Omar H Chai WJ Idris Z. Alteration in the functional Organization of the Default Mode Network Following Closed non-severe Traumatic Brain Injury. Front Neurosci. (2022) 16:833320. doi: 10.3389/fnins.2022.833320, PMID: 35418832 PMC8995774

[23] Monroe DC Blumenfeld RS Keator DB Solodkin A Small SL. One season of head-to-ball impact exposure alters functional connectivity in a central autonomic network. NeuroImage. (2020) 223:117306. doi: 10.1016/j.neuroimage.2020.117306, PMID: 32861790 PMC7822072

[24] Walton SR Powell JR Brett BL Yin W Kerr ZY Liu M. Associations of lifetime concussion history and repetitive head impact exposure with resting-state functional connectivity in former collegiate American football players: an NCAA 15-year follow-up study. PLoS One. (2022) 17:e0273918. doi: 10.1371/journal.pone.0273918, PMID: 36084077 PMC9462826

[25] Wong JKY Churchill NW Graham SJ Baker AJ Schweizer TA. Altered connectivity of default mode and executive control networks among female patients with persistent post-concussion symptoms. Brain Inj. (2023) 37:147–58. doi: 10.1080/02699052.2022.2163290, PMID: 36594665

[26] Zhou Y Milham MP Lui YW Miles L Reaume J Sodickson DK. Default-mode network disruption in mild traumatic brain injury. Radiology. (2012) 265:882–92. doi: 10.1148/radiol.12120748, PMID: 23175546 PMC3504316

[27] Zhu DC Covassin T Nogle S Doyle S Russell D Pearson RL. A potential biomarker in sports-related concussion: brain functional connectivity alteration of the default-mode network measured with longitudinal resting-state fMRI over thirty days. J Neurotrauma. (2015) 32:327–41. doi: 10.1089/neu.2014.3413, PMID: 25116397

[28] Cassoudesalle H Petit A Chanraud S Petit H Badaut J Sibon I. Changes in resting-state functional brain connectivity associated with head impacts over one men’s semi-professional soccer season. J Neurosci Res. (2021) 99:446–54. doi: 10.1002/jnr.24742, PMID: 33089563

[29] Champagne AA Coverdale NS Nashed JY Fernandez-Ruiz J Cook DJ. Resting CMRO(2) fluctuations show persistent network hyper-connectivity following exposure to sub-concussive collisions. NeuroImage Clin. (2019) 22:101753. doi: 10.1016/j.nicl.2019.101753, PMID: 30884366 PMC6424143

[30] DeSimone JC Davenport EM Urban J Xi Y Holcomb JM Kelley ME. Mapping default mode connectivity alterations following a single season of subconcussive impact exposure in youth football. Hum Brain Mapp. (2021) 42:2529–45. doi: 10.1002/hbm.25384, PMID: 33734521 PMC8090779

[31] Johnson B Neuberger T Gay M Hallett M Slobounov S. Effects of subconcussive head trauma on the default mode network of the brain. J Neurotrauma. (2014) 31:1907–13. doi: 10.1089/neu.2014.3415, PMID: 25010992 PMC4238241

[32] Manning KY Brooks JS Dickey JP Harriss A Fischer L Jevremovic T. Longitudinal changes of brain microstructure and function in nonconcussed female rugby players. Neurology. (2020) 95:E402–12. doi: 10.1212/WNL.0000000000009821, PMID: 32554762 PMC7455316

[33] Militana AR Donahue MJ Sills AK Solomon GS Gregory AJ Strother MK. Alterations in default-mode network connectivity may be influenced by cerebrovascular changes within 1 week of sports related concussion in college varsity athletes: a pilot study. Brain Imaging Behav. (2016) 10:559–68. doi: 10.1007/s11682-015-9407-3, PMID: 25972119 PMC4644725

[34] Monroe DC DuBois SL Rhea CK Duffy DM. Age-related trajectories of brain structure-function coupling in female roller Derby athletes. Brain Sci. (2022) 12. doi: 10.3390/brainsci12010022, PMID: 35053766 PMC8774127

[35] Murugesan G Saghafi B Davenport E Wagner B Urban J Kelley M. Single season changes in resting state network Power and the connectivity between regions: distinguish head impact exposure level in high school and youth football players. Proc SPIE Int Soc Opt Eng. (2018) 10575:105750F. doi: 10.1117/12.2293199 PMC6884358 31787799

[36] Slobounov SM Walter A Breiter HC Zhu DC Bai X Bream T. The effect of repetitive subconcussive collisions on brain integrity in collegiate football players over a single football season: a multi-modal neuroimaging study. NeuroImage Clin. (2017) 14:708–18. doi: 10.1016/j.nicl.2017.03.006, PMID: 28393012 PMC5377433

[37] Wilson A Stevens WD Sergio L Wojtowicz M. Altered brain functional connectivity in female athletes over the course of a season of collision or contact sports. Neurotrauma Rep. (2022) 3:377–87. doi: 10.1089/neur.2022.0010, PMID: 36204391 PMC9531888

[38] Siddiqi SH Kandala S Hacker CD Trapp NT Leuthardt EC Carter AR. Individualized precision targeting of dorsal attention and default mode networks with rTMS in traumatic brain injury-associated depression. Sci Rep. (2023) 13:4052. doi: 10.1038/s41598-022-21905-x, PMID: 36906616 PMC10008633

[39] Baron SL Hein MJ Lehman E Gersic CM. Body mass index, playing position, race, and the cardiovascular mortality of retired professional football players. Am J Cardiol. (2012) 109:889–96. doi: 10.1016/j.amjcard.2011.10.050, PMID: 22284915

[40] Lehman EJ Hein MJ Baron SL Gersic CM. Neurodegenerative causes of death among retired National Football League players. Neurology. (2012) 79:1970–4. doi: 10.1212/WNL.0b013e31826daf50, PMID: 22955124 PMC4098841

[41] Lee TA Lycke RJ Lee PJ Cudal CM Torolski KJ Bucherl SE. Distribution of head acceleration events varies by position and play type in north American football. Clin J Sport Med. (2021) 31:E245–50. doi: 10.1097/JSM.0000000000000778, PMID: 32032162

[42] Vike N Bari S Susnjar A Lee T Lycke R Auger J. American football position-specific Neurometabolic changes in high school athletes: a magnetic resonance spectroscopic study. J Neurotrauma. (2022) 39:1168–82. doi: 10.1089/neu.2021.0186, PMID: 35414265

[43] Krill MK Borchers JR Hoffman JT Krill ML Hewett TE. Analysis of football injuries by position Group in Division I College Football: a 5-year program review. Clin J Sport Med. (2020) 30:216–23. doi: 10.1097/JSM.0000000000000574, PMID: 32341288

[44] Nathanson JT Connolly JG Yuk F Gometz A Rasouli J Lovell M. Concussion incidence in professional football: position-specific analysis with use of a novel metric. Orthop J Sports Med. (2016) 4:2325967115622621. doi: 10.1177/2325967115622621, PMID: 26848481 PMC4731682

[45] Powell JW Barber-Foss KD. Traumatic brain injury in high school athletes. JAMA. (1999) 282:958–63. doi: 10.1001/jama.282.10.958, PMID: 10485681

[46] Kerr ZY Wilkerson GB Caswell SV Currie DW Pierpoint LA Wasserman EB. The first decade of web-based sports injury surveillance: descriptive epidemiology of injuries in United States high school football (2005–2006 through 2013–2014) and National Collegiate Athletic Association Football (2004–2005 through 2013–2014). J Athl Train. (2018) 53:738–51. doi: 10.4085/1062-6050-144-17, PMID: 30138047 PMC6188086

[47] Papa L Walter AE Wilkes JR Clonts HS Johnson B Slobounov SM. Effect of player position on serum biomarkers during participation in a season of collegiate football. J Neurotrauma. (2022) 39:1339–48. doi: 10.1089/neu.2022.0083, PMID: 35615873 PMC9529311

[48] Jones CMA Kamintsky L Parker E Kureshi N Audas L Wilson L. Blood–brain barrier dysfunction and exposure to head impacts in university football players. Clin J Sport Med. (2024) 34:61–8. doi: 10.1097/JSM.0000000000001164, PMID: 37285595

[49] Horstemeyer MF Berthelson PR Moore J Persons AK Dobbins A Prabhu RK. A mechanical brain damage framework used to model abnormal brain tau protein accumulations of national football league players. Ann Biomed Eng. (2019) 47:1873–88. doi: 10.1007/s10439-019-02294-1, PMID: 31372858 PMC6757135

[50] Alsalaheen B Stockdale K Pechumer D Giessing A He X Broglio SP. Cumulative effects of concussion history on baseline computerized neurocognitive test scores: systematic review and Meta-analysis. Sports Health. (2017) 9:324–32. doi: 10.1177/1941738117713974, PMID: 28661827 PMC5496709

[51] Chizuk HM Cunningham A Horn EC Thapar RS Willer BS Leddy JJ. Association of Concussion History and Prolonged Recovery in youth. Clin J Sport Med Off J Can Acad Sport Med. (2022) 32:e573–9. doi: 10.1097/JSM.0000000000001044, PMID: 35533140 PMC9633345

[52] Howell DR Beasley M Vopat L Meehan WP. The effect of prior concussion history on dual-task gait following a concussion. J Neurotrauma. (2017) 34:838–44. doi: 10.1089/neu.2016.4609, PMID: 27541061

[53] Churchill N Hutchison MG Leung G Graham S Schweizer TA. Changes in functional connectivity of the brain associated with a history of sport concussion: a preliminary investigation. Brain Inj. (2017) 31:39–48. doi: 10.1080/02699052.2016.1221135, PMID: 27901587

[54] Esteban O Markiewicz CJ Blair RW Moodie CA Isik AI Erramuzpe A. fMRIPrep: a robust preprocessing pipeline for functional MRI. Nat Methods. (2019) 16:111–6. doi: 10.1038/s41592-018-0235-4, PMID: 30532080 PMC6319393

[55] Behzadi Y Restom K Liau J Liu TT. A component based noise correction method (CompCor) for BOLD and perfusion based fMRI. NeuroImage. (2007) 37:90–101. doi: 10.1016/j.neuroimage.2007.04.042, PMID: 17560126 PMC2214855

[56] Muschelli J Nebel MB Caffo BS Barber AD Pekar JJ Mostofsky SH. Reduction of motion-related artifacts in resting state fMRI using aCompCor. NeuroImage. (2014) 96:22–35. doi: 10.1016/j.neuroimage.2014.03.028, PMID: 24657780 PMC4043948

[57] Power JD Mitra A Laumann TO Snyder AZ Schlaggar BL Petersen SE. Methods to detect, characterize, and remove motion artifact in resting state fMRI. NeuroImage. (2014) 84:320–41. doi: 10.1016/j.neuroimage.2013.08.048, PMID: 23994314 PMC3849338

[58] Schaefer A Kong R Gordon EM Laumann TO Zuo XN Holmes AJ. Local-global Parcellation of the human cerebral cortex from intrinsic functional connectivity MRI. Cereb Cortex. (2018) 28:3095–114. doi: 10.1093/cercor/bhx179, PMID: 28981612 PMC6095216

[59] Fox MD Raichle ME. Spontaneous fluctuations in brain activity observed with functional magnetic resonance imaging. Nat Rev Neurosci. (2007) 8:700–11. doi: 10.1038/nrn2201, PMID: 17704812

[60] Franco AR Pritchard A Calhoun VD Mayer AR. Interrater and intermethod reliability of default mode network selection. Hum Brain Mapp. (2009) 30:2293–303. doi: 10.1002/hbm.20668, PMID: 19206103 PMC2751639

[61] Greicius MD Srivastava G Reiss AL Menon V. Default-mode network activity distinguishes Alzheimer’s disease from healthy aging: evidence from functional MRI. Proc Natl Acad Sci USA. (2004) 101:4637–42. doi: 10.1073/pnas.0308627101, PMID: 15070770 PMC384799

[62] Johnson B Dodd A Mayer AR Hallett M Slobounov S. Are there any differential responses to concussive injury in civilian versus athletic populations: a neuroimaging study. Brain Imaging Behav. (2020) 14:110–7. doi: 10.1007/s11682-018-9982-1, PMID: 30361946

[63] Sharma B Nowikow C DeMatteo C Noseworthy MD Timmons BW. Sex-specific differences in resting-state functional brain activity in pediatric concussion. Sci Rep. (2023) 13:3284. doi: 10.1038/s41598-023-30195-w, PMID: 36841854 PMC9968337

[64] Meier TB Bellgowan PSF Mayer AR. Longitudinal assessment of local and global functional connectivity following sports-related concussion. Brain Imaging Behav. (2017) 11:129–40. doi: 10.1007/s11682-016-9520-y, PMID: 26821253

[65] Brett BL Bryant AM España LY Mayer AR Meier TB. Investigating the overlapping associations of prior concussion, default mode connectivity, and executive function-based symptoms. Brain Imaging Behav. (2022) 16:1275–83. doi: 10.1007/s11682-021-00617-2, PMID: 34989980 PMC9107488

[66] Garcia-Cordero I Vasilevskaya A Taghdiri F Khodadadi M Mikulis D Tarazi A. Functional connectivity changes in neurodegenerative biomarker-positive athletes with repeated concussions. J Neurol. (2024) 271:4180–90. doi: 10.1007/s00415-024-12340-1, PMID: 38589629

[67] Elkin BS Gabler LF Panzer MB Siegmund GP. Brain tissue strains vary with head impact location: a possible explanation for increased concussion risk in struck versus striking football players. Clin Biomech Bristol Avon. (2019) 64:49–57. doi: 10.1016/j.clinbiomech.2018.03.021, PMID: 29625747

[68] Utevsky AV Smith DV Huettel SA. Precuneus is a functional Core of the default-mode network. J Neurosci. (2014) 34:932–40. doi: 10.1523/JNEUROSCI.4227-13.2014, PMID: 24431451 PMC3891968

[69] Yang Z Chang C Xu T Jiang L Handwerker DA Castellanos FX. Connectivity trajectory across lifespan differentiates the precuneus from the default network. NeuroImage. (2014) 89:45–56. doi: 10.1016/j.neuroimage.2013.10.039, PMID: 24287438 PMC3944140

[70] Cavanna AE Trimble MR. The precuneus: a review of its functional anatomy and behavioural correlates. Brain J Neurol. (2006) 129:564–83. doi: 10.1093/brain/awl004, PMID: 16399806

[71] Thomas Yeo BT Krienen FM Sepulcre J Sabuncu MR Lashkari D Hollinshead M. The organization of the human cerebral cortex estimated by intrinsic functional connectivity. J Neurophysiol. (2011) 106:1125–65. doi: 10.1152/jn.00338.2011, PMID: 21653723 PMC3174820

[72] Le TH Pardo JV Hu X. 4 T-fMRI study of nonspatial shifting of selective attention: cerebellar and parietal contributions. J Neurophysiol. (1998) 79:1535–48. doi: 10.1152/jn.1998.79.3.1535, PMID: 9497430

[73] Simon O Mangin JF Cohen L Le Bihan D Dehaene S. Topographical layout of hand, eye, calculation, and language-related areas in the human parietal lobe. Neuron. (2002) 33:475–87. doi: 10.1016/S0896-6273(02)00575-5, PMID: 11832233

[74] Krause BJ Schmidt D Mottaghy FM Taylor J Halsband U Herzog H. Episodic retrieval activates the precuneus irrespective of the imagery content of word pair associates. A PET study. Brain. (1999) 122:255–63. doi: 10.1093/brain/122.2.255, PMID: 10071054

[75] Daneshvar DH Nair ES Baucom ZH Rasch A Abdolmohammadi B Uretsky M. Leveraging football accelerometer data to quantify associations between repetitive head impacts and chronic traumatic encephalopathy in males. Nat Commun. (2023) 14:3470. doi: 10.1038/s41467-023-39183-0, PMID: 37340004 PMC10281995

[76] Papa L Slobounov SM Breiter HC Walter A Bream T Seidenberg P. Elevations in MicroRNA biomarkers in serum are associated with measures of concussion, neurocognitive function, and subconcussive trauma over a single National Collegiate Athletic Association Division I Season in collegiate football players. J Neurotrauma. (2019) 36:1343–51. doi: 10.1089/neu.2018.6072, PMID: 30343622 PMC6909756

[77] Omalu BI DeKosky ST Minster RL Kamboh MI Hamilton RL Wecht CH. Chronic traumatic encephalopathy in a National Football League player. Neurosurgery. (2005) 57:128, 128–34. doi: 10.1227/01.NEU.0000163407.92769.ED, PMID: 15987548

[78] National Collegiate Athletics Association. Year-round football practice contact for college student-athletes recommendations [internet]. National Collegiate Athletics Association; (2016). Available at:https://ncaaorg.s3.amazonaws.com/ssi/concussion/SSI_YearRoundFootballPracticeContactRecommendations.pdf

