# Supplementary material for: Association of player position and functional connectivity alterations in collegiate American football players: an fMRI study
Source: Front Neurol. 2025 Jan 7;15:1511915. doi: 10.3389/fneur.2024.1511915 (PMC11776490; doi:10.3389/fneur.2024.1511915)
Supplement: Supplementary file 1 [file Table_1.DOCX]

**Supplemental Figure 1**. Creation of individual functional connectivity maps for the DMN. **(A)** The posterior cingulate cortex was pre- selected as the seed ROI from which to carry out functional analysis with all other voxels of the brain. Mean time series correlation coefficients between the PCC and the whole brain were calculated for pre- and post-season. Blue represents negative correlation coefficients, while yellow-red represents positive correlation coefficients. Then negative coefficients were removed for pre- and post-season, leaving only regions with a positive correlation. **(B)** The regions with positive correlation coefficients were mapped on to 10 regions of the DMN from the Schaefer-Yeo anatomical atlas (Schaefer et al., 2018). The ten regions designated from this atlas included the left and right: parietal (Par), temporal (Temp), ventral prefrontal cortex (PFCv), precuneus posterior cingulate cortex (pCunPCC), dorsal medial prefrontal cortex (PFCdPFCm).

**Supplemental Figure 2. Pre-season and post-season comparison of FC of 10 DMN ROIs. (A)** Pre-season compared with post-season functional connectivity of the left hemisphere DMN ROIs. **(B)** Pre-season compared with post-season FC of PCC to right hemisphere DMN ROIs. **(C)** Illustration of the total number of subjects that demonstrated increased (purple) and decreased (green) functional connectivity from pre-season to post-season in each ROI.

**Supplemental Figure 3. Pre-season and post-season functional connectivity comparison for Non-Speed players. (A)** Pre-season compared with post-season functional connectivity of PCC to the left hemisphere DMN ROIs in Non-Speed players. **(B)** Pre-season compared with post-season FC of PCC to right hemisphere DMN ROIs in Non-Speed players. **(C)** Illustration of the total number of subjects that demonstrated increased (purple) and decreased (green) functional connectivity from pre-season to post-season in each ROI. **(D)** Box-and-whisker plots showing alterations in functional connectivity from pre-season to post-season, via Z-values of correlation coefficient from paired sample t-tests of post-season minus that of pre-season for all 10 DMN ROIs. Blue boxes show decreases in hypoconnectivity (decreased functional connectivity) from pre-season to post-season, while Red boxes show hyperconnectivity (increased functional connectivity) from pre-season to post-season.

**Supplemental Figure 4. Pre-season and post-season functional connectivity comparison for Speed players. (A)** Pre-season compared with post-season functional connectivity of PCC to the left hemisphere DMN ROIs in Speed players. **(B)** Pre-season compared with post-season FC of PCC to right hemisphere DMN ROIs in Speed players. **(C)** Illustration of the total number of subjects that demonstrated increased (purple) and decreased (green) functional connectivity from pre-season to post-season in each ROI. **(D)** Box-and-whisker plots showing alterations in functional connectivity from pre-season to post-season, via Z-values of correlation coefficient from paired sample t-tests of post-season minus that of pre-season for all 10 DMN ROIs. Blue boxes show decreases in hypoconnectivity (decreased functional connectivity) from pre-season to post-season, while Red boxes show hyperconnectivity (increased functional connectivity) from pre-season to post-season.

**Supplemental Figure 5. Pre-season and post-season functional connectivity comparison for players with no history of concussion. (A)** Pre-season compared with post-season functional connectivity of PCC to the left hemisphere DMN ROIs in players with no history of concussion. **(B)** Pre-season compared with post-season FC of PCC to right hemisphere DMN ROIs in players with no history of concussion. **(C)** Illustration of the total number of subjects that demonstrated increased (purple) and decreased (green) functional connectivity from pre-season to post-season in each ROI. **(D)** Box-and-whisker plots showing alterations in functional connectivity from pre-season to post-season, via Z-values of correlation coefficient from paired sample t-tests of post-season minus that of pre-season for all 10 DMN ROIs. Blue boxes show decreases in hypoconnectivity (decreased functional connectivity) from pre-season to post-season, while Red boxes show hyperconnectivity (increased functional connectivity) from pre-season to post-season.

**Supplemental Figure 6. Pre-season and post-season functional connectivity comparison for players with history of concussion (Cx-History). (A)** Pre-season compared with post-season functional connectivity of PCC to the left hemisphere DMN ROIs in players with Cx-History. **(B)** Pre-season compared with post-season FC of PCC to right hemisphere DMN ROIs in players with Cx-History. **(C)** Illustration of the total number of subjects that demonstrated increased (purple) and decreased (green) functional connectivity from pre-season to post-season in each ROI. **(D)** Box-and-whisker plots showing alterations in functional connectivity from pre-season to post-season, via Z-values of correlation coefficient from paired sample t-tests of post-season minus that of pre-season for all 10 DMN ROIs. Blue boxes show decreases in hypoconnectivity (decreased functional connectivity) from pre-season to post-season, while Red boxes show hyperconnectivity (increased functional connectivity) from pre-season to post-season.
