# Supplementary figures and images for: Association of player position and functional connectivity alterations in collegiate American football players: an fMRI study
Source: Front Neurol. 2025 Jan 7;15:1511915. doi: 10.3389/fneur.2024.1511915 (PMC11776490; doi:10.3389/fneur.2024.1511915)

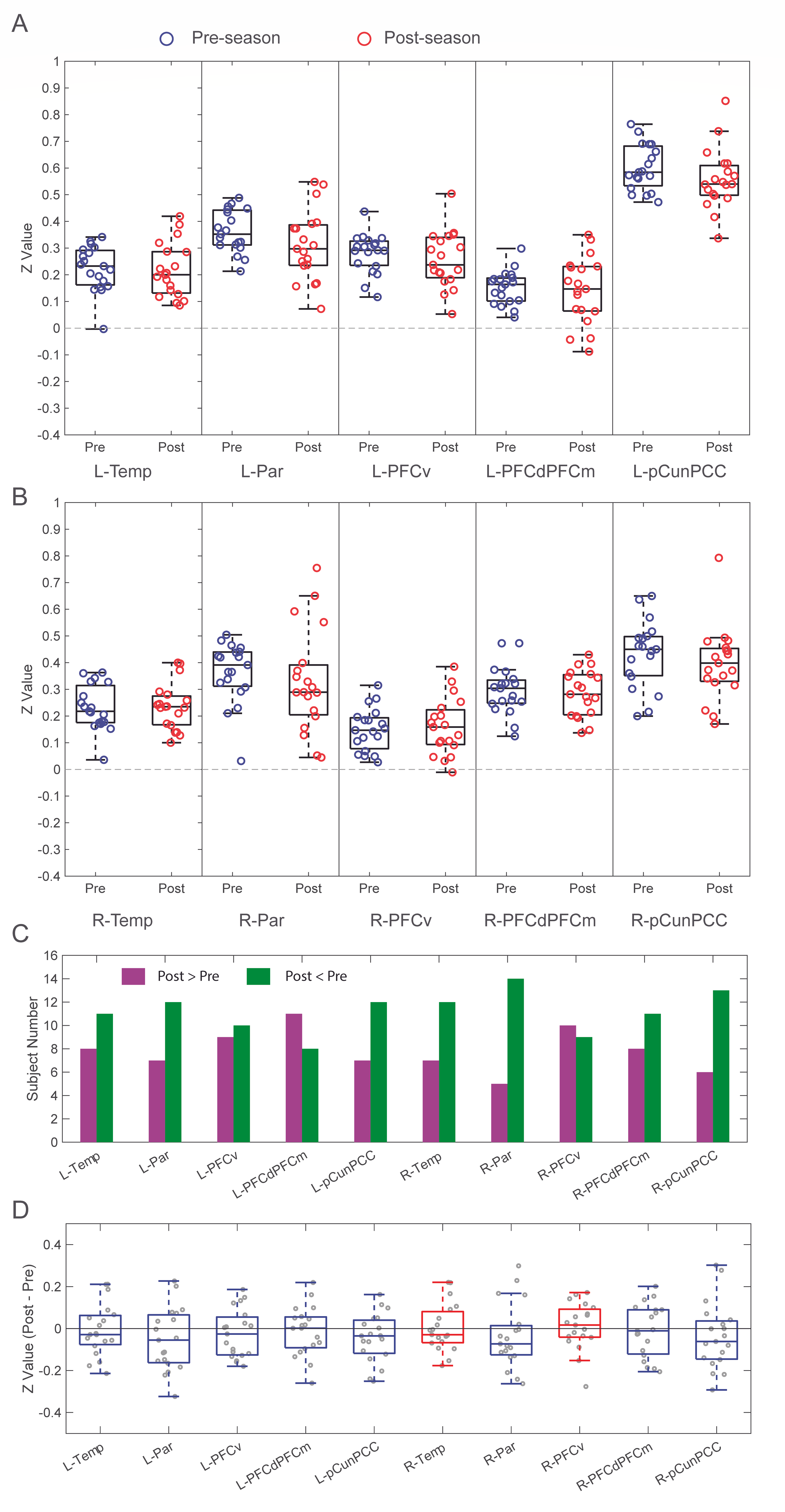

Supplement: Supplementary file 2 [file Image_1.TIFF]

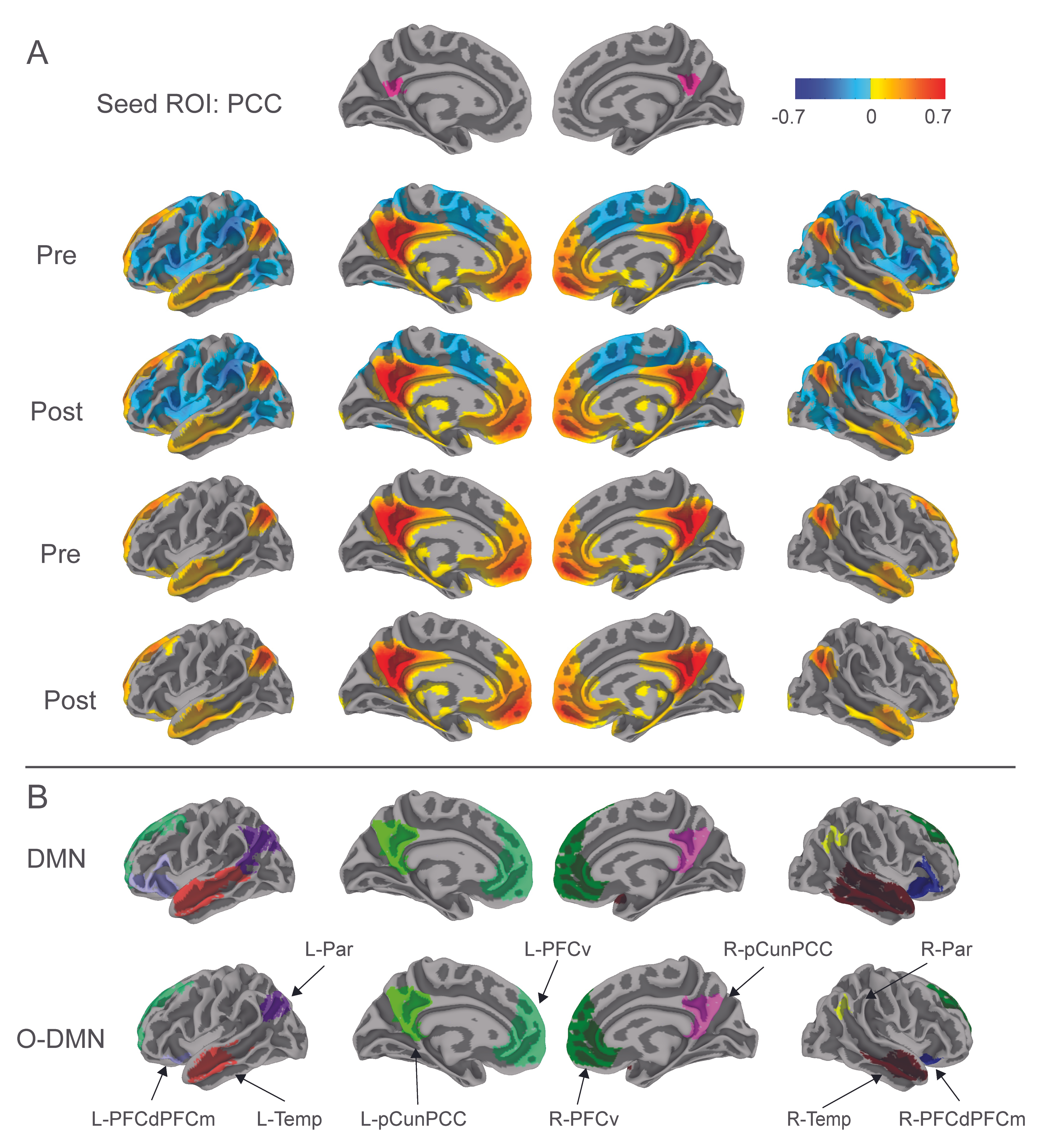

Supplement: Supplementary file 3 [file Image_2.TIFF]

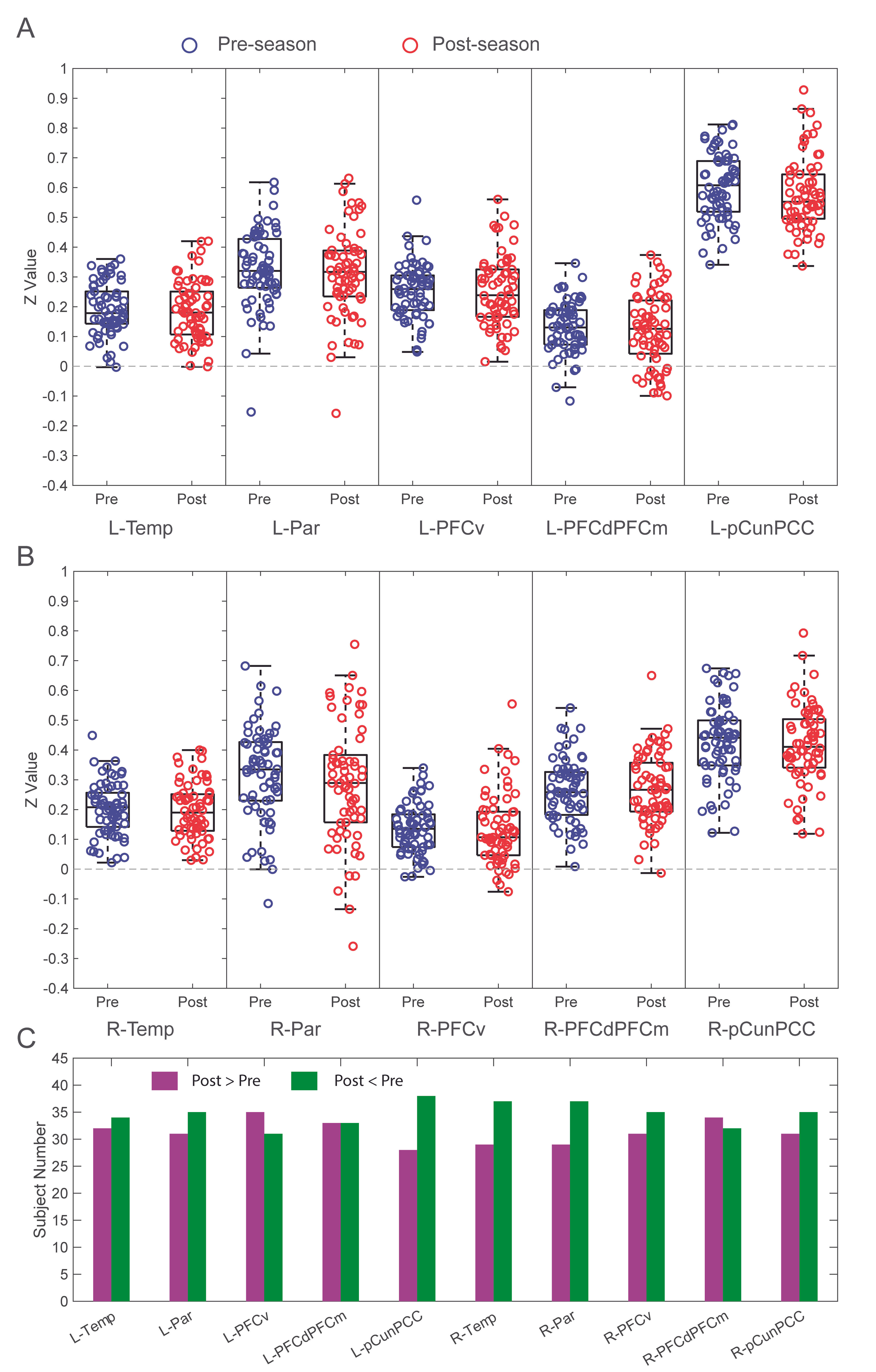

Supplement: Supplementary file 4 [file Image_3.TIFF]

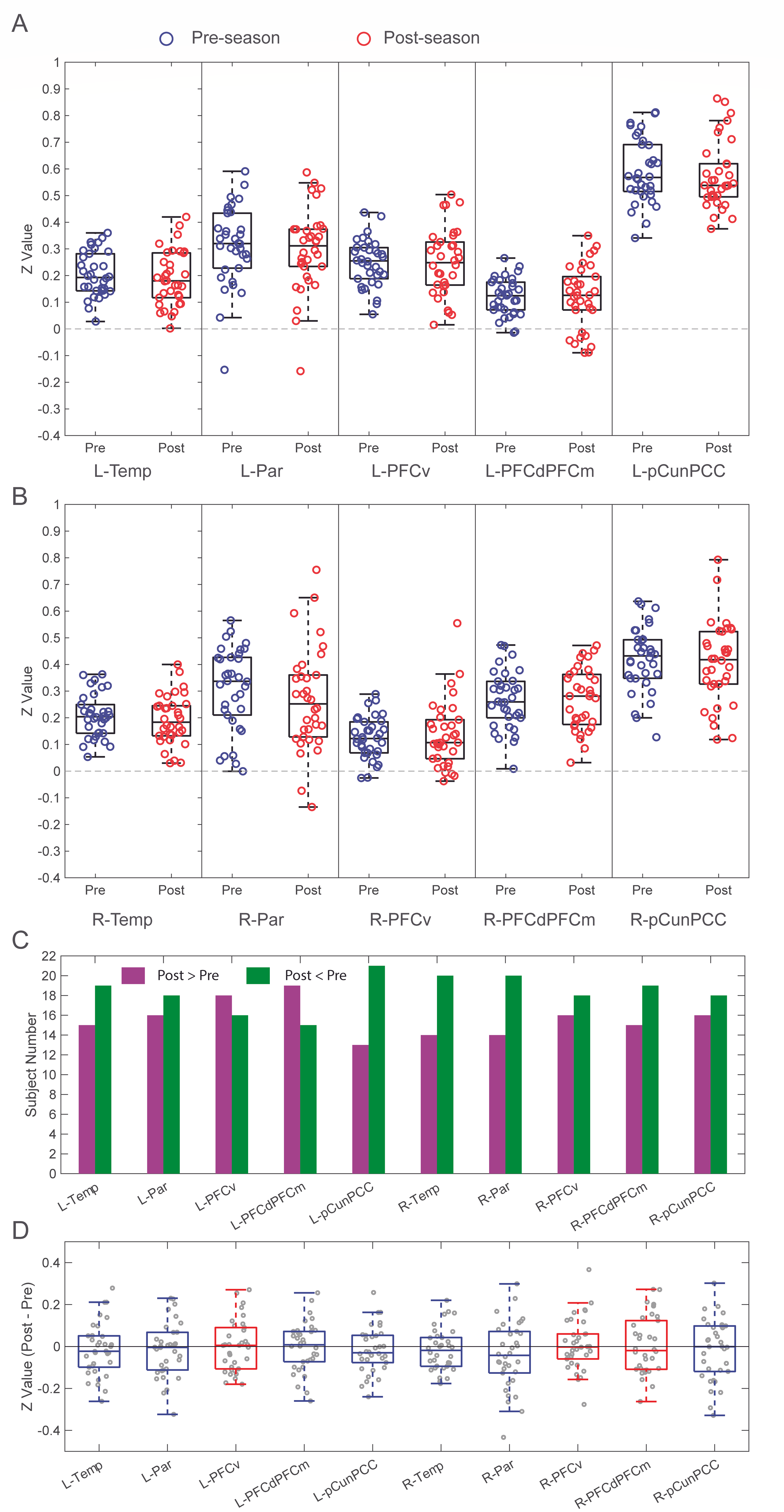

Supplement: Supplementary file 5 [file Image_4.TIFF]

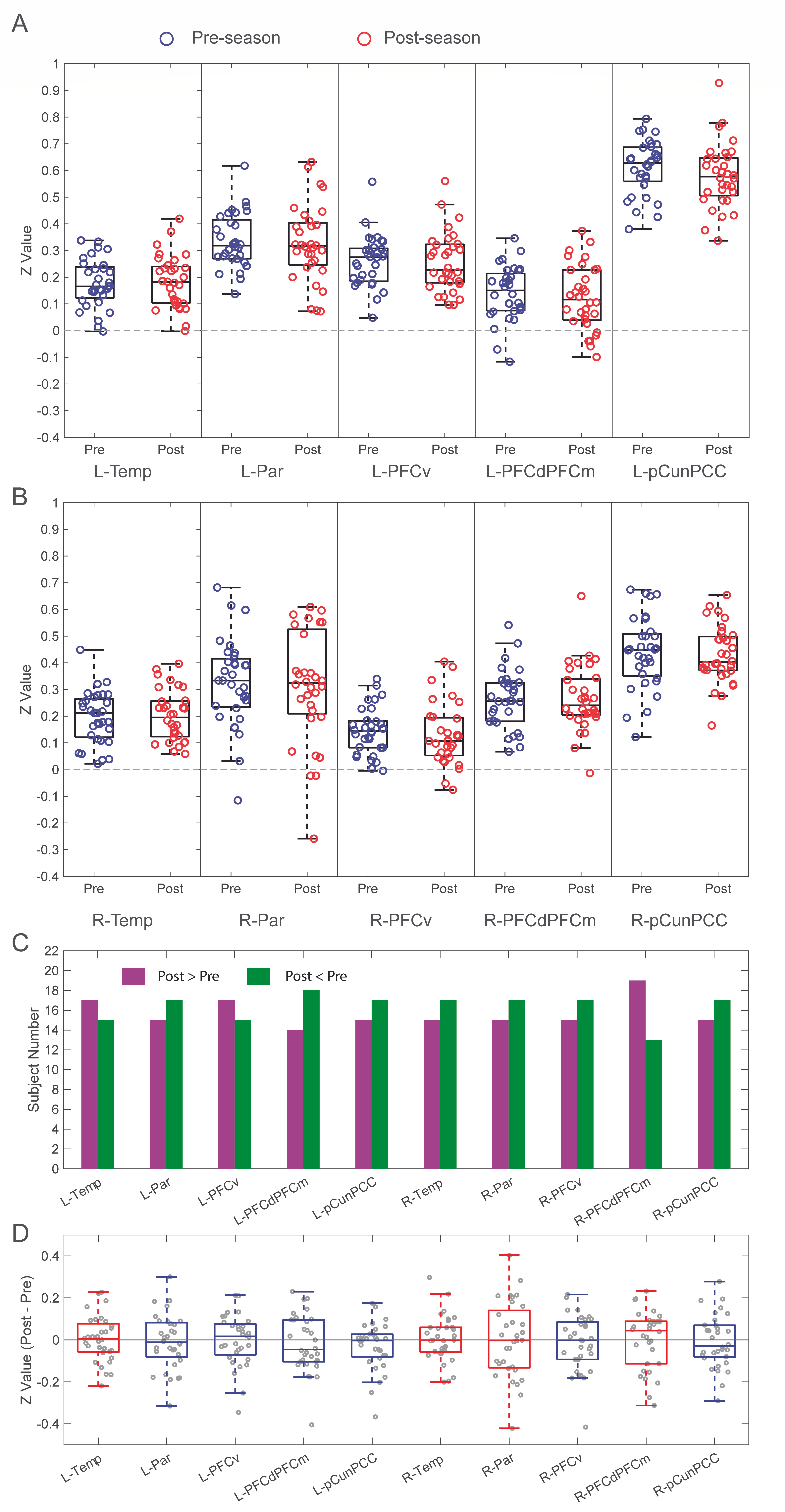

Supplement: Supplementary file 6 [file Image_5.TIFF]

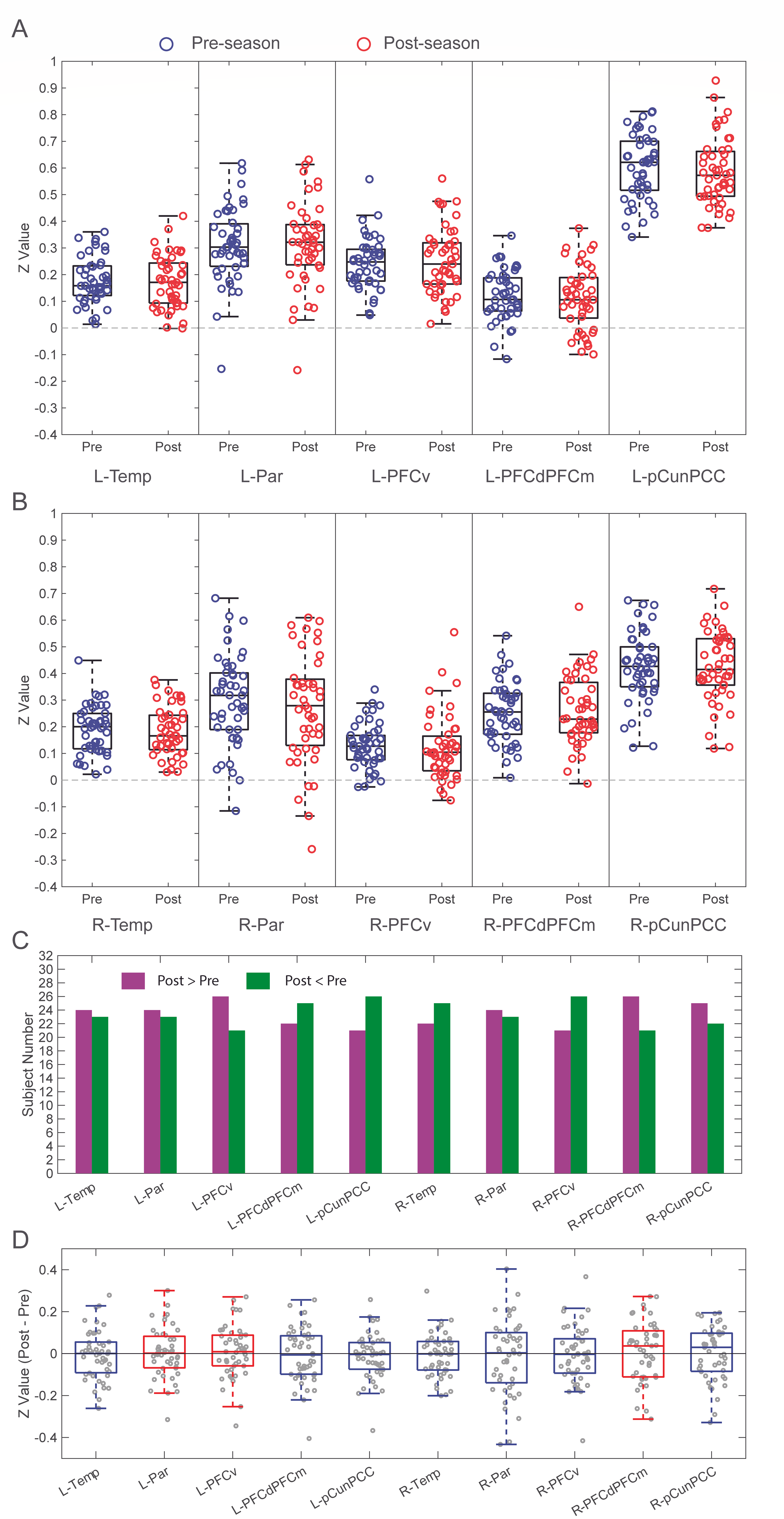

Supplement: Supplementary file 7 [file Image_6.TIFF]
